# Bridging Inflammation and Oncology: The Role and Therapeutic Potential of Macrophage Migration Inhibitory Factor in Lung Cancer

**DOI:** 10.3390/ijms27062829

**Published:** 2026-03-20

**Authors:** Mohammed Ali Selo, Oliviero L. Gobbo, Ismael Obaidi, Christine O’Connor, Darren Fayne, Michelle E. Armstrong, Seamas C. Donnelly

**Affiliations:** 1 School of Medicine, Trinity Biomedical Sciences Institute (TBSI), Trinity College Dublin, D02 R590 Dublin, Ireland; obaidi.ismael@gmail.com (I.O.); michelle.armstrong@tcd.ie (M.E.A.); 2 Faculty of Pharmacy, University of Kufa, Najaf 54001, Iraq; 3 School of Pharmacy and Pharmaceutical Sciences, Panoz Institute, Trinity College Dublin, D02 PN40 Dublin, Ireland; ogobbo@tcd.ie; 4 Trinity St. James’s Cancer Institute, St. James’s Hospital, D08 NHY1 Dublin, Ireland; 5 Faculty of Pharmacy, University of Babylon, Babylon 51002, Iraq; 6 Discipline of Clinical Medicine, Trinity Centre for Health Sciences, Tallaght University Hospital, D24 NR0A Dublin, Ireland; christine.oconnor@tuh.ie; 7 School of Chemical Sciences, DCU Life Sciences Institute, Dublin City University, D09 V209 Glasnevin, Ireland; darren.p.fayne@dcu.ie

**Keywords:** tumor, tautomerase, macrophage migration inhibitory factor (MIF) polymorphisms, CATT repeats, targeted therapy, oxidized MIF (oxMIF), metastasis, survival rate

## Abstract

Lung cancer is the leading cause of cancer-related mortality worldwide, accounting for more deaths than any other malignancy. Despite advances in treatment, it remains highly lethal, with 5-year survival rates showing minimal improvement over the past several decades, highlighting a critical unmet clinical need. Macrophage Migration Inhibitory Factor (MIF) is a multifunctional cytokine that contributes to inflammation and cancer, promoting tumor growth, progression, and metastasis through modulation of the tumor microenvironment, stimulation of angiogenesis, and regulation of immune responses. Polymorphisms in the promoter region of MIF, such as high-expression CATT repeats, influence MIF expression and susceptibility to a range of inflammatory, autoimmune, and malignant disorders, yet their role in lung cancer remains largely unexplored. Therapeutic strategies targeting MIF, including small-molecule inhibitors, antibodies, and peptide-based agents, have shown promise in preclinical models, although their clinical translation is still limited. This review discusses the dual role of MIF in inflammation and oncology, summarizes current therapeutic developments, and emphasizes the potential of MIF-targeted interventions in lung cancer. It discusses the significance of genetic predisposition, particularly high-expression MIF alleles, in guiding personalized treatment strategies for lung cancer and identifying patients who may derive benefit from MIF inhibition.

## 1. Introduction

Lung cancer is one of the most aggressive and lethal malignancies worldwide, consistently ranking among the leading cancers in both incidence and mortality. It has remained the leading cause of cancer-related deaths worldwide since 1985, with both incidence and mortality steadily rising, and the high fatality rate causing mortality to mirror incidence [1]. Globally, in 2008, it accounted for 1.6 million new cases (13% of all cancers) and 1.4 million deaths (18% of cancer-related deaths), ranking first in incidence and mortality among men and fourth in incidence and second in mortality among women [2]. This upward trend continued with 1.8 million cases and 1.6 million deaths in 2012 [3] and 2.09 million cases and 1.76 million deaths in 2018 [4,5]. Although breast cancer briefly surpassed lung cancer in incidence in 2020, lung cancer remained the leading cause of cancer-related mortality with 1.8 million deaths [6]. By 2022, lung cancer had regained its position as the most commonly diagnosed cancer worldwide, with nearly 2.5 million new cases (12.4%), and remained the leading cause of cancer mortality with over 1.8 million deaths (18.7%) [7]. If current trends persist, global lung cancer burden is projected to reach 4.62 million cases and 3.55 million deaths by 2050 [8]. Overall, between 2008 and 2022, lung cancer accounted for approximately 12.4–13% of all cancer cases and 18–18.7% of cancer-related deaths worldwide. Thus, it remains the deadliest malignancy, causing more deaths than any other cancer; for example, in the United States, lung cancer mortality equaled the combined deaths from prostate, colon, breast, and pancreatic cancers in 2011 [9,10] and continued to exceed the combined deaths from breast, colorectal, and prostate cancers in 2022 [11].

Lung cancer is broadly categorized into two major histological types, small cell lung cancer (SCLC) and non-small cell lung cancer (NSCLC). NSCLC accounts for approximately 85–90% of all cases [12] and comprises several subtypes, including adenocarcinoma, squamous cell carcinoma, and large cell carcinoma [13,14]. Treatment strategies are stage-dependent, with early-stage NSCLC (I and II) typically managed through surgical resection, often followed by adjuvant therapy, while advanced or metastatic disease (III and IV) is primarily treated with chemotherapy and/or radiotherapy [15]. However, conventional chemotherapies are limited by non-specificity, low bioavailability, and drug resistance. To address these issues, newer approaches such as nanocarrier-based delivery systems, molecular targeted therapies, photothermal therapy, and immunotherapy have emerged [12]. Despite these advances, prognosis remains poor, with lung cancer still showing one of the lowest 5-year survival rates among all cancers [5] and survival rates have shown minimal improvement over the past several decades [16]. In this context, global data from 1995 to 2014 across approximately 70 countries showed that lung cancer remains highly lethal, with age-standardized 5-year net survival consistently low, fluctuating between 10–20% in both developed and developing regions [17,18]. During this period, survival rates remained largely unchanged, with improvements limited to 5–10% in 21 countries and exceeding 10% in only three countries, highlighting the minimal improvement in lung cancer survival rates [17]. More recent analyses, covering data from 1990 to 2018, indicate that this pattern has changed very little, with global 5-year relative or net survival remaining in the range of 10–20% and remaining substantially lower than that observed for all other major cancers [19]. For instance, in the United States, the 5-year overall survival for lung cancer was only 15.6% between 2001 and 2007 and remained among the lowest at 19% during 2008–2014, compared with much higher rates for other cancers such as prostate (98%), melanoma (92%), and breast (90%) [20].

Given the substantial clinical burden of lung cancer, understanding the molecular mechanisms underlying its onset and progression is essential for developing novel and targeted therapies. The disease is highly complex and heterogeneous, driven by extensive genomic diversity, a broad mutation spectrum, and cumulative genetic and epigenetic changes that disrupt tumor suppressors and activate oncogenic pathways [21,22,23]. Among these, p53 is the most frequently mutated tumor suppressor, and its alterations are associated with poor prognosis, resistance to DNA-damaging therapies, and limited response to immunotherapy, highlighting the need for novel therapeutic strategies specifically targeting mutant p53 [24,25]. Other commonly affected tumor suppressors include retinoblastoma and the p16 pathway [26], while major oncogenic drivers include the rat sarcoma (RAS) and members of the human epidermal growth factor receptor (HER) family [27]. Tumor progression also relies on angiogenesis, driven mainly by vascular endothelial growth factor (VEGF), which enables growth beyond 3 mm by establishing a vascular network for nutrient and oxygen supply [23,28]. Notably, many of these pathways are influenced by macrophage migration inhibitory factor (MIF) (see Section 5 and Section 6).

Building on the evidence discussed above, lung cancer remains a major therapeutic challenge and a significant unmet clinical need, with persistently high incidence, mortality, and minimal improvements in survival despite advances in diagnostics and treatment. This review therefore focuses on MIF and its role in inflammatory diseases and cancer, with particular emphasis on its regulation of key molecular, inflammatory, and immune pathways in lung cancer, as well as emerging evidence linking MIF genetic polymorphisms to cancer susceptibility and its potential as a therapeutic target.

## 2. Overview of Macrophage Migration Inhibitory Factor (MIF)

MIF is a multifunctional, pleiotropic cytokine that plays a central role in immune response and inflammation. It was first identified for its ability to inhibit the migration of immune cells, particularly macrophages—hence its name [29]. MIF was first identified by Bloom and Bennett in 1966, when they described the phenomenon of migration inhibition during studies on delayed-type hypersensitivity, characterizing it as a soluble factor produced by sensitized lymphocytes that suppresses macrophage migration in vitro in response to specific antigens [29]. The cloning of human MIF cDNA in 1989 [30] enabled detailed exploration of its biological activities, revealing macrophages as both a source and target of MIF [31]. By 1993, MIF was recognized as a clinically relevant pro-inflammatory cytokine that potentiates lethal endotoxemia [32], sparking extensive research into its roles in immune regulation, inflammation, and receptor-mediated signaling, and leading to the identification of its molecular targets and disease-modifying effects [33].

MIF is expressed by a broad range of immune and non-immune cell types in both in vitro and in vivo settings [34]. It is constitutively present in many cells but can be further upregulated by inflammatory, hypoxic, or tumor-associated signals, as detailed in Table 1, which summarizes its reported cellular sources and regulatory contexts.

### 2.1. Structure and Isoforms

MIF has a homotrimer structure composed of three identical 114–amino-acid subunits, arranged with three-fold rotational symmetry around a central solvent-accessible channel. Each monomer contains two antiparallel α-helices and a four-stranded β-sheet, and the central channel has been proposed as a binding site for small molecules such as glutathione, gangliosides, or dopachrome [52]. Its evolutionary conservation across plants, protozoans, nematodes, and other invertebrates underscores its fundamental biological importance [53].

Interestingly, increasing evidence indicates that MIF exists in two post-translational redox forms, oxidized and reduced [54]. This redox interconversion is regulated by redox-sensitive residues within its structure, particularly lysine-66 and cysteine-80 (the “switch cysteine”) [55]. The oxidized MIF (oxMIF) predominates in inflammatory and malignant conditions, while the reduced form is mainly present in healthy tissues, suggesting that oxMIF represents the active, disease-associated isoform [56,57] (see Section 4 and Section 5). Notably, oxidation at specific residues can also reduce MIF’s biological activity; for instance, oxidation of the N-terminal proline (Pro-1) impairs its tautomerase activity and ability to bind and activate CD74 signaling [58], suggesting that selective Pro-1 oxidation could represent a potential strategy to attenuate MIF’s pro-tumorigenic effects. Thus, the precise role of oxMIF in disease progression and its functional implications require further investigation. Clarifying how redox modifications influence MIF’s activity and signaling pathways may reveal new opportunities for therapeutic intervention in inflammatory and malignant diseases.

### 2.2. Signaling

Upon secretion, MIF exerts its effects through both autocrine and paracrine signaling, primarily via binding to its primary receptor CD74. This interaction forms a functional complex with the co-receptor CD44, which is essential for full activation of downstream pathways. The MIF–CD74–CD44 complex triggers both transient and sustained activation of the extracellular signal-regulated kinase 1/2 (ERK1/2) mitogen-activated protein kinase (MAPK) cascade via Src family tyrosine kinase signaling [59]. This leads to nuclear factor kappa B (NF-κB) translocation to the nucleus, upregulation of phospholipase A2 and prostaglandin synthesis, and stimulation of the arachidonic acid pathway [60], events which subsequently activate c-Jun N-terminal kinase (JNK) and enhance the translation of tumor necrosis factor-alpha (TNF-α) and other cytokine mRNAs [61]. In addition to CD74-dependent signaling, MIF can also interact with non-canonical C-X-C chemokine receptors (CXCR) including CXCR2 and CXCR4, thereby amplifying its role in inflammatory, autoimmune, and neoplastic processes [62]. Collectively, these receptor interactions highlight the complexity of MIF signaling and its broad implications in human pathophysiology.

### 2.3. Catalytic Activities

MIF exhibits unique enzymatic activities, functioning as a D-dopachrome tautomerase [63], a phenylpyruvate tautomerase [64], and a thiol-protein oxidoreductase [65], with the latter contributing to redox homeostasis, defense against oxidative stress and apoptosis inhibition [66]. MIF’s enzymatic activity may also exert a neuroprotective effect by detoxifying catecholamine breakdown products, converting toxic quinone derivatives into less harmful dihydroxyindoles, which serve as precursors of neuromelanin [67]. A study using tautomerase-null MIF knock-in mice has shown that MIF’s catalytic Pro1 residue is essential for many of its biological effects and may play a role in mediating crucial protein–protein interactions [68,69]. The finding that MIF interacts with its CD74 receptor near its tautomerase site suggested that small-molecule inhibitors targeting this region could disrupt the MIF–CD74 interaction, thereby blocking this key protein–protein interaction essential to MIF’s function [70,71]. To facilitate the identification of such inhibitors, a biochemical assay was developed to evaluate compounds for inhibition of the MIF–CD74 protein–protein interaction [70]. A study by El-Turk et al. proposed that targeting MIF’s C-terminal region could offer novel approaches for allosteric modulation of its enzymatic activity and aid in the development of new inhibitors of MIF tautomerase activity [72].

Many studies have shown that inhibition of MIF-tautomerase activity may represent a therapeutic approach for various inflammatory diseases and malignancies. For example, in a murine sepsis model, inhibition of MIF’s tautomerase activity with ISO-1, a widely used small-molecule MIF inhibitor commercially available for experimental studies, significantly reduced both lethality and TNF-α levels, highlighting the MIF active site as a potential therapeutic target for treating sepsis [73]. In a murine model of chronic *Pseudomonas aeruginosa* infection, we found that mice lacking MIF tautomerase activity (mif^P1G/P1G^) exhibited significantly reduced lung inflammation, lower bacterial burden, and less tissue damage compared with wild-type animals [74]. SCD-19, a novel small-molecule tautomerase inhibitor identified by our group that completely abolishes MIF enzymatic activity and is more effective than ISO-1, markedly reduced pulmonary inflammation in the same model [75], highlighting the role of MIF’s tautomerase activity in driving pulmonary inflammation in cystic fibrosis. The small molecule 4-iodo-6-phenylpyrimidine (4-IPP) was shown to irreversibly bind to MIF’s catalytic N-terminal proline, thereby inhibiting its enzymatic activity and more effectively suppressing MIF-induced lung adenocarcinoma cell migration and growth than ISO-1 [76]. Our group further demonstrated that MIF’s tautomerase activity is critical for lung cancer progression as tumor growth was markedly reduced in MIF-knockout and enzymatically inactive MIF^P1G/P1G^ mice [77]. In both a murine Lewis Lung Carcinoma (LLC) cell line and in tumor-bearing mice, SCD-19 more potently suppressed cell proliferation and tumor growth than ISO-1, reducing primary tumor volume in mice by approximately 90% [77].

Altogether, MIF’s tautomerase activity contributes to its biological functions in both inflammatory and oncogenic contexts. Experimental studies indicate that inhibition of MIF’s catalytic activity attenuates downstream signaling, inflammatory responses, and tumor cell proliferation in preclinical models. Notably, in lung cancer models, pharmacologic and genetic inhibition of this enzymatic activity suppresses tumor growth and cellular proliferation, supporting its functional relevance in lung tumor progression. However, while preclinical data supports its importance in lung cancer models, further studies are needed to clarify the precise molecular mechanisms linking tautomerase activity to receptor signaling and downstream oncogenic pathways in human lung malignancies. These findings also support continued investigation of selective small-molecule inhibitors targeting this catalytic domain as a potential therapeutic strategy in lung cancer.

## 3. MIF Promoter Polymorphisms and Their Clinical Implications

**Figure 1:**
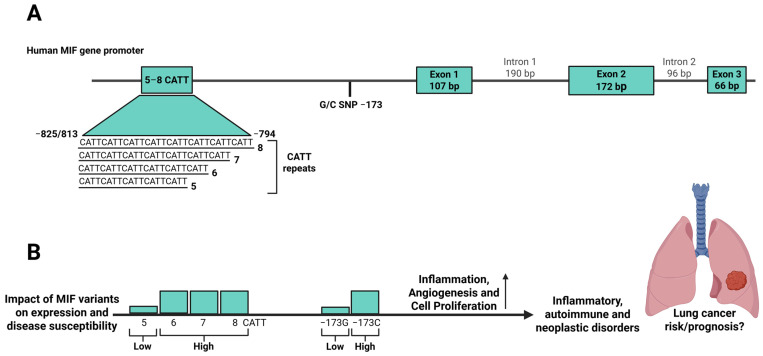
Schematic representation of the human *macrophage migration inhibitory factor* (*MIF)* gene and its promoter polymorphisms. (**A**) The human *MIF* gene is composed of three exons and two introns. Two functional polymorphisms in the promoter region, a tetranucleotide repeat (−794 CATT5–8) and a single-nucleotide variant (−173 G/C), modulate *MIF* transcriptional activity. Created with BioRender.com based on concepts described in Reference [85]. (**B**) Longer CATT6–8 repeats and the −173 C allele are associated with increased *MIF* expression, which promote inflammatory responses, angiogenesis and cell proliferation and predispose individuals to inflammatory, autoimmune and malignant disorders and may also be relevant to lung cancer susceptibility or prognosis, which remains to be investigated in lung cancer cohorts. Created in BioRender. Selo, M. A. (2026) https://BioRender.com/gqd3pdi, accessed on 5 December 2025.

The human *MIF* gene is located on chromosome 22 (22q11.2) and is composed of three exons of 107, 172, and 66 base pairs (bp) and two introns of 188 and 94 bp (see Figure 1) [44,78]. Genetic variations within the *MIF* promoter region have been shown to influence its transcriptional regulation. Specifically, two functional polymorphisms have been identified, a functional tetranucleotide CATT5–8 repeat at −794 (rs5844572) and a G/C single-nucleotide polymorphism (SNP) at −173 (rs755622) [79]. Both variants are associated with differential expression. With respect to the −173 G/C SNP, the −173 C allele enhances binding of the activator protein 4 (*AP4*) transcription factor, leading to increased transcriptional activity. Both in vitro and in vivo studies have demonstrated that this allele correlates with increased *MIF* gene and protein expression levels [79,80]. Similarly, the −794 CATT5–8 tetranucleotide repeat modulates *MIF* promoter activity in a length-dependent manner, with the CATT5 allele showing the lowest promoter activity [81], while longer repeats (CATT6–8) are associated with elevated *MIF* expression [82,83]. This is mediated by the transcription factor ICBP90, which binds to the CATT5–8 microsatellite region, where longer repeats strengthen the interaction and enhance MIF transcription [84], (see Figure 1).

Both −173 G/C and −794 CATT5–8 MIF polymorphisms, particularly alleles associated with higher MIF expression, have been linked to susceptibility and severity of various autoimmune, inflammatory, and neoplastic conditions. A comprehensive meta-analysis of 96 case–control studies involving thousands of participants showed that the MIF −173 G/C polymorphism is strongly associated with increased risk of autoimmune-inflammatory, infectious, and age-related diseases across diverse global populations, with significant associations in Asian, European, and Latin American cohorts [86]. A subsequent meta-analysis of 23 studies further confirmed this association with autoimmune disorders [87]. This −173 G/C variant has also been linked to cardiovascular diseases [88,89], increased susceptibility to pulmonary tuberculosis, particularly among Asians [90] and, based on a meta-analysis of ten studies involving 2203 cancer cases and 2805 controls, with overall cancer risk [91]. However, none of these studies specifically examined the potential link of the variant with lung cancer.

Likewise, accumulating evidence indicates that the CATT5–8 polymorphism contributes to the susceptibility and severity of inflammatory and autoimmune diseases, commonly alongside the −173 G/C variant. In rheumatoid arthritis (RA), the low-expressing CATT5 allele was associated with milder [81], while higher-expressing alleles, particularly CATT7, were correlated with elevated circulating MIF levels and more severe, erosive joint damage [80]. A more recent meta-analysis of 12 studies confirmed that the −173 C allele, the CATT7 repeat, and the CATT7–MIF −173 C haplotype were linked to increased RA susceptibility across multiple ethnicities [92]. Similarly, a meta-analysis of 13 studies revealed higher circulating MIF levels in systemic lupus erythematosus (SLE) patients, particularly among Asians, with the −173 C/G polymorphism, but not −794 CATT, linked to disease susceptibility [93]. Paradoxically, in a cohort of 1369 SLE patients, the high-expression −794 CATT7/−173 C haplotype was associated with lower SLE incidence, yet CATT5 alleles were less frequent in those with organ damage, indicating that high-expression CATT6–8 alleles contributed to more severe organ involvement [83]. In cystic fibrosis, CATT5 was associated with milder disease, lower rates of *Pseudomonas* colonization [94], and a slower forced expiratory volume in 1 s (FEV1) decline [74].

The variants have also been implicated in cancer, with the high-expression −173 C allele and longer CATT6–8 repeats associated with increased risk of rectal [95] and non-cardia gastric cancer, particularly among *Helicobacter pylori*-infected individuals [96]. The CATT7 repeat was further linked to early-stage cervical cancer [97] and to higher prostate cancer incidence and recurrence [98]. In cutaneous squamous cell carcinoma, the MIF 5C (−794 CATT5/−173 C) and 7G (−794 CATT7/−173 G) haplotypes conferred higher disease susceptibility, accompanied by elevated circulating MIF levels [99].

To further investigate the functional impact of MIF promoter polymorphisms, our group developed novel humanized mouse models carrying the low-expressing CATT5 and high-expressing CATT7 human MIF alleles [100], providing an important experimental platform to elucidate how MIF genetic variation influences disease susceptibility and pathogenesis. The high-expression −794 CATT7 allele was linked to more severe COVID-19, with hospitalized patients exhibiting elevated serum MIF levels, and mice carrying this allele developing more severe disease than CATT5 carriers [100]. In preclinical asthma models, CATT7 mice showed pronounced airway inflammation than CATT5 or wild-type mice, which was effectively attenuated by the MIF inhibitor SCD-19 [101]. Moreover, mesenchymal stromal cells and SCD-19 treatment effectively attenuated airway inflammation and MIF-dependent macrophage priming in high-MIF CATT7 mice, further supporting the role of high-expression (CATT6–8) MIF alleles in asthma severity [102,103]. Similarly, a recent study using the YUMMER1.7 murine melanoma model demonstrated that tumors grew faster in high-MIF CATT7 mice, whereas CATT5 carriers exhibited a one week delay in tumor establishment and significantly reduced tumor burden by day 15 [104]. A comprehensive summary of diseases linked to MIF promoter polymorphisms, highlighting their associations with susceptibility and severity, is provided in Table 2.

Overall, high-expression promoter variants of MIF (i.e., the −173 C allele and longer −794 CATT6–8 repeats) are frequently associated with greater disease susceptibility and/or severity across a variety of inflammatory, autoimmune, infectious and malignant disorders. These findings underscore not only the broad mechanistic importance of MIF in disease pathogenesis through immune activation, tissue injury, and tumor-promoting effects, but also highlight the translational potential of MIF-targeted therapies, particularly for individuals with high-expression alleles who may derive the greatest benefit from anti-MIF interventions. Importantly, the contribution of these promoter polymorphisms to lung cancer susceptibility or progression has not yet been directly examined. Given the multiple oncogenic pathways in which MIF has been implicated, determining whether high-expression MIF alleles influence lung cancer risk, disease behavior, or treatment response remains to be investigated in lung cancer cohorts. At present, lung cancer–specific genotype–phenotype correlation data remain scarce, and prospective clinical studies will be necessary to clarify the potential prognostic and predictive significance of these variants in this setting. These considerations highlight key unanswered questions and underscore the need for rigorous lung-focused validation.

## 4. MIF and Inflammatory Diseases

MIF is a key proinflammatory mediator that amplifies inflammatory responses both directly by stimulating the release of proinflammatory cytokines, and indirectly by counteracting the anti-inflammatory effects of glucocorticoids [114,115,116]. Administration of anti-MIF antibodies has been shown to completely protect adrenalectomized rodents from lethal arthritis, highlighting MIF’s role in amplifying inflammatory pathways when not counterbalanced by endogenous glucocorticoids [117]. As discussed previously in Section 3, the identification of functional polymorphisms in the MIF promoter region, which are associated with several inflammatory disorders, has further underscored MIF’s significance in the pathogenesis of inflammatory diseases [118]. Indeed, MIF has been implicated in a wide number of immune and inflammatory diseases. For example, in acute respiratory distress syndrome, MIF has been shown to be a key driver of the harmful inflammatory response which can be reduced by anti-MIF treatment [115]. Likewise, elevated MIF levels were detected in the bronchoalveolar lavage fluid of asthmatic patients compared to non-atopic individuals [119] and MIF-deficient mice exhibited reduced pulmonary inflammation and airway hyperresponsiveness following ovalbumin challenge compared to wild-type animals [120]. Recently, MIF has also been implicated in severe neutrophilic asthma, which is often resistant to glucocorticoid therapy, by promoting neutrophil recruitment to the lungs and reducing glucocorticoid effectiveness through inhibition of annexin-A1 [121], and to promote airway remodeling in addition to its established role in inflammation [122].

In cystic fibrosis, MIF and its tautomerase activity, which drive pulmonary inflammation and earlier *Pseudomonas* colonization, have been linked to disease severity [74,94]. Furthermore, *P. aeruginosa* has been shown to exploit recombinant human MIF (rhMIF) to enhance biofilm formation, and rhMIF was found to impair the antibacterial efficacy of tobramycin in vitro [75].

Further evidence of MIF’s role in inflammatory pathogenesis comes from its involvement in autoimmune and systemic inflammatory diseases. In RA, MIF is associated with increased susceptibility [105], disease severity [80,81,123], and a higher risk of developing inflammatory polyarthritis [106]. In SLE, MIF and its oxidized form are elevated [93], with higher circulating [124], and urinary [125] concentrations correlating with disease activity and inflammatory organ involvement [83,126]. Plasma oxMIF rises during systemic flares, whereas urinary oxMIF increases specifically in lupus nephritis, reflecting local renal inflammation and disease severity [57]. Elevated MIF and oxMIF have also been detected in inflamed tissues of patients with Crohn’s disease and ulcerative colitis as well as in the plasma of individuals with severe sepsis and septicemia [57,127], further underscoring MIF’s central role in systemic inflammation.

Taken together, these findings highlight MIF as a central mediator of inflammatory and immune-driven pathology across diverse diseases. By promoting tissue inflammation, impairing glucocorticoid responsiveness, and enhancing immune cell recruitment, MIF contributes to disease progression in asthma, chronic pulmonary infections such as cystic fibrosis, and autoimmune conditions including SLE and RA. Importantly, its enzymatic and signaling functions position MIF as a promising therapeutic target, with anti-MIF strategies offering potential benefits for steroid-resistant, treatment-refractory, or genetically high-risk patient populations. Beyond inflammatory disorders, these MIF-mediated immune-modulatory and pro-inflammatory mechanisms are also broadly relevant to oncogenesis, including lung cancer, where related signaling pathways contribute to tumor progression and microenvironmental remodeling (see Section 5 and Section 6).

## 5. MIF and Cancer

MIF has emerged as a key regulator of tumor biology, promoting cancer progression through diverse mechanisms, including tumor growth, angiogenesis, and modulation of the immune microenvironment. The following subsections summarize the molecular mechanisms of MIF, its impact on anti-tumor immunity, and its clinical associations across cancer types.

### 5.1. Molecular Mechanisms of MIF-Driven Tumorigenesis

The link between inflammation and cancer has long been recognized, with chronic inflammatory diseases associated with increased risk of various malignancies [63,128]. While inflammatory pathways normally protect against infection and injury, they can also create a tumor-promoting microenvironment that supports growth and metastasis [129]. In this context, MIF acts as a molecular bridge between inflammation and tumorigenesis by sustaining chronic inflammatory signaling and promoting tumor-supportive conditions [63]. Through engagement of its receptors, MIF activates downstream signaling pathways that promote tumor growth and survival. It downregulates p53 and inhibits its nuclear localization, thereby impairing apoptotic responses and facilitating tumor cell survival [130,131,132,133].

MIF further contributes to tumor progression by promoting angiogenesis. It stimulates endothelial cell migration and vascular differentiation through MAPK- and phosphatidylinositol-3-kinase/protein kinase B (PI3K/Akt)-dependent upregulation of VEGF [134,135], thereby facilitating neovascularization and tumor growth. Within hypoxic tumor environments, MIF engages in a positive feedback loop with hypoxia-inducible factor 1α (HIF-1α), reinforcing transcriptional programs that promote angiogenesis, glycolysis, invasion, and survival [42,136,137]. MIF also promotes metastatic progression by inducing epithelial-to-mesenchymal transition (EMT) [138], and upregulating matrix metalloproteinase 9 [139], thereby facilitating extracellular matrix remodeling and tumor invasion.

The tautomerase active site of MIF is also critical for its oncogenic function, as tumor growth was markedly reduced in tautomerase-null (MIF^P1G/P1G^) mice in both skin [68] and lung cancer [77] models, highlighting the importance of this structural region in tumor progression [68,133]. Consistently, pharmacological inhibition of this site with SCD-19 suppressed lung cancer growth in vitro and in vivo [77].

**Figure 2:**
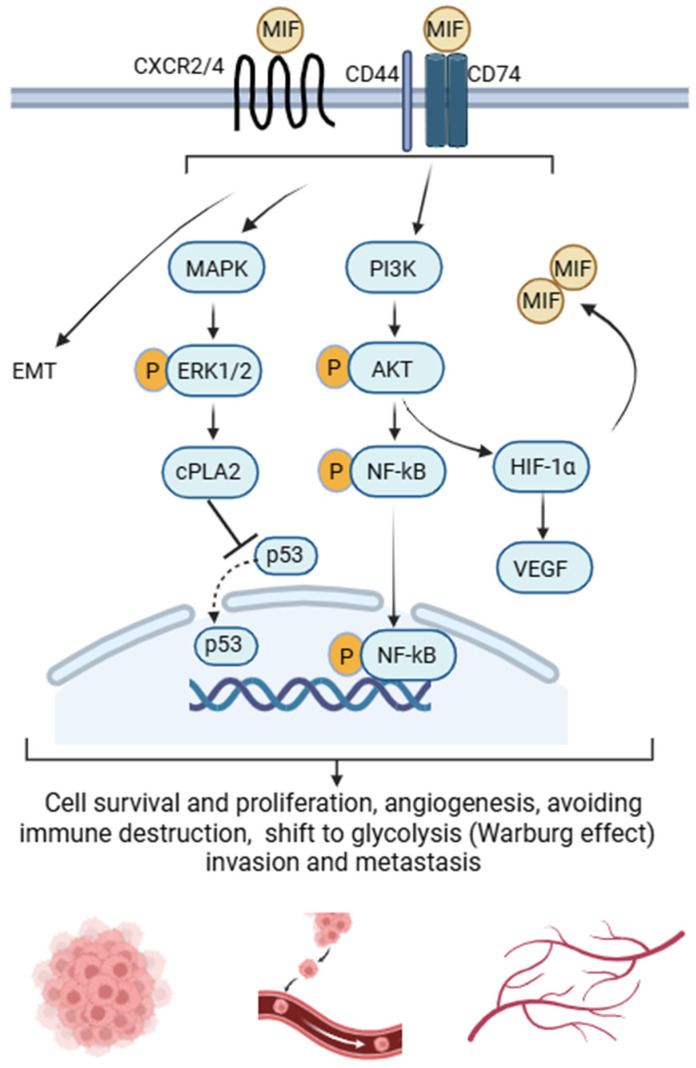
Schematic representation of macrophage migration inhibitory factor (MIF)-mediated signaling in tumor progression. MIF binds to the CD74/CD44 complex or CXC chemokine receptors 2 and 4 (CXCR2/4), thereby activating downstream mitogen-activated protein kinase–extracellular signal-regulated kinase 1/2 (MAPK–ERK1/2) and phosphatidylinositol 3-kinase/protein kinase B (PI3K/Akt) signaling pathways. ERK1/2 signaling stimulates cytosolic phospholipase A2 (cPLA2) and inhibits nuclear translocation of p53, promoting tumor cell survival and proliferation. Concurrently, PI3K/Akt activation induces nuclear factor kappa B (NF-κB)-mediated transcription of proinflammatory cytokines and vascular endothelial growth factor (VEGF), driving inflammation and angiogenesis. MIF also promotes epithelial–mesenchymal transition (EMT) and stabilizes hypoxia-inducible factor 1α (HIF-1α), which further enhances MIF and VEGF expression and drives a metabolic shift toward aerobic glycolysis (the Warburg effect). This establishes a positive feedback loop that supports tumor growth, angiogenesis, invasion, and metastatic progression. Created in BioRender. Selo, M. A. (2026) https://BioRender.com/f0f538a, accessed on 2 December 2025.

Collectively, these findings position MIF as a central regulator of tumor progression, linking inflammatory and stress-associated signaling to cancer cell survival, angiogenesis, invasion, and resistance to cell death. The major molecular pathways contributing to these effects are summarized in Figure 2.

### 5.2. MIF–CD74 Signaling and Resistance to Immune Checkpoint Blockade

The MIF–CD74 axis plays a crucial role in promoting immunosuppression across multiple cancers by shaping the tumor microenvironment. MIF–CD74 interaction activates downstream pathways that polarize tumor-associated macrophages toward an M2 immunosuppressive phenotype and limit infiltration and activity of cytotoxic CD8^+^ T cells [40,140]. In Ewing’s sarcoma, this leads to impaired antitumor immunity, while inhibition of MIF–CD74 reprograms the microenvironment, enhances T-cell activity, and suppresses tumor growth [41]. Similarly, in metastatic melanoma, blocking MIF–CD74 signaling on macrophages and dendritic cells reduces immunosuppressive factor expression and enhances cytotoxic T-cell activation, restoring antitumor immune responses [40].

Mechanistically, MIF–CD74 interaction directly regulates programmed death-ligand 1 (PD-L1) expression on tumor cells, a key immune checkpoint molecule targeted by programmed cell death protein 1 (PD-1)/PD-L1 inhibitors. High CD74/PD-L1 expression correlates with immune evasion in melanoma models, providing a mechanistic basis for resistance to checkpoint blockade [141]. Inhibiting the MIF–CD74 axis increases T-cell infiltration, shifts macrophages toward a pro-inflammatory phenotype, and reduces PD-L1 expression in tumor cells, effectively enhancing innate immune activation and sensitizing tumors to immunotherapy [140]. In addition to its role in immune checkpoint regulation, MIF–CD74 signaling also influences responses to other therapies through remodeling of the tumor microenvironment. In NSCLC brain metastases, inhibition of the MIF–CD74 axis enhanced radiotherapy efficacy by reversing radiation-induced Akt phosphorylation and promoting polarization of microglia toward a pro-inflammatory M1 phenotype [47]. Although this study did not directly assess immune checkpoint inhibitors, it highlights the broader impact of MIF–CD74-mediated innate immune reprogramming on therapeutic responses.

Consistent with these observations, combining MIF-targeted therapies with PD-1/PD-L1 blockade potentially represents a promising strategy to overcome resistance in cancers characterized by MIF-driven immunosuppressive microenvironments. For example, in preclinical YUMMER1.7 melanoma and MC38 colorectal carcinoma mouse models, dual targeting of MIF and PD-1 enhanced antitumor responses, reduced tumor growth, prolonged survival, and promoted more complete tumor regression with enhanced intratumoral immune activation compared with anti-PD-1 or anti-MIF monotherapy [104].

Taken together, these studies identify the MIF–CD74 axis as a key regulator of tumor immune suppression and therapeutic resistance. By modulating macrophage polarization, T-cell infiltration, and PD-L1 expression, MIF–CD74 signaling may limit the efficacy of immune checkpoint blockade. PD-1/PD-L1 inhibitors have become an important component of cancer immunotherapy, with clinical activity reported across numerous solid tumors, highlighting the broad relevance of this therapeutic axis in oncology [142,143,144]. In lung cancer, particularly NSCLC, immune checkpoint inhibitors now constitute a central component of therapy across advanced and earlier-stage disease, including neoadjuvant and adjuvant settings, with demonstrated survival benefit in selected patients [145,146]. Nevertheless, resistance remains a limitation to durable clinical benefit [145]. These observations support further investigation of combination strategies integrating MIF-targeted therapies with immune checkpoint blockade, including in lung cancer models where MIF-driven immune modulation may influence therapeutic responsiveness. If validated, such approaches could inform future translational and clinical development.

### 5.3. MIF Overexpression and Clinical Associations Across Cancer Types

MIF is overexpressed in many types of cancer, and elevated expression has been linked to aggressive tumor phenotypes and poorer clinical outcomes across multiple malignancies [147,148]. A systematic meta-analysis of multiple solid tumors demonstrated that high MIF expression correlates with poorer overall survival and disease-free survival in cancer patients, supporting its prognostic relevance across diverse cancer types [149]. In line with these findings, pan-cancer analyses show that MIF is upregulated across numerous malignancies and is associated with genomic instability, immune suppression, and other features of aggressive disease [150]. While lung cancer is the primary focus of this review, selected evidence from other malignancies is mentioned below to demonstrate the broader clinical implications of MIF overexpression. Comprehensive pan-cancer and prognostic analyses are provided in other systematic reviews and meta-analyses [149,150].

Elevated MIF levels have been reported in colorectal cancer, where increased expression is observed in primary tumors, metastatic lesions, and patient serum, with higher circulating MIF concentrations correlating with disease severity and metastatic burden [139]. Similarly, MIF has been shown to be involved in the initiation and progression of head and neck cancers, where it suppresses apoptosis and facilitates both local and distant metastases and thereby contribute to poor prognosis [151]. Epithelial-derived MIF was shown to drive colorectal cancer progression and maintenance, highlighting its potential as a therapeutic target, with inhibition potentially enhancing anticancer therapy [152].

In addition to total MIF, the disease-associated oxMIF has been detected at elevated levels in several malignancies, including colorectal, pancreatic, ovarian, and lung cancers [57,153]. Within the tumor microenvironment, MIF oxidation appears to promote tumor growth and invasion [56,153]. The clinical relevance of targeting oxMIF is supported by early-phase evaluation of the monoclonal antibody imalumab in patients with advanced solid tumors [154]. Consistently, preclinical studies demonstrate that targeting oxMIF with monoclonal antibodies induces apoptosis, suppresses tumor cell proliferation, and enhances the efficacy of chemotherapeutic agents in both in vitro and in vivo cancer models [153,155].

Overall, MIF overexpression is a common feature across diverse malignancies and is closely associated with aggressive tumor behavior and poor clinical outcomes, highlighting its potential as both a biomarker and a therapeutic target in cancer.

## 6. MIF and Lung Cancer

As mentioned previously in Section 1 and Section 5, MIF is involved in many of the signaling pathways that are involved in lung cancer pathogenesis. Although MIF has been implicated in several inflammatory and malignant diseases, its specific therapeutic potential in lung cancer remains insufficiently explored. In vitro studies consistently demonstrate that MIF overexpression promotes NSCLC progression through autocrine and paracrine mechanisms, activating multiple oncogenic pathways such as MAPK and PI3K/Akt [43,48,131]. Elevated MIF mRNA and protein levels have been observed in several lung adenocarcinoma cell lines compared with normal primary alveolar epithelial cells [156]. Functionally, MIF overexpression enhances proliferation, migration, invasion, adhesion, and spreading of lung cancer cells, whereas MIF knockdown or pharmacological inhibition markedly suppresses these processes [43,49,157]. Mechanistically, MIF overexpression promoted lung cancer cell proliferation and the Warburg effect via NF-κB-dependent HIF-1α upregulation [158], while MIF silencing in H460 cells markedly reduced proliferation and triggered apoptosis through activation of caspases-3 and -4 [159]. Moreover, MIF promoted angiogenesis in NSCLC by inducing C-X-C motif chemokine ligand 8 (CXCL8) and VEGF expression through a CD74-dependent JNK/activator protein-1 (AP-1) signaling pathway [160].

In silico analyses have further supported these findings, identifying MIF as part of a 4-gene signature that stratifies lung adenocarcinoma patients into high- and low-risk groups for overall and recurrence-free survival, with markedly lower 5-year recurrence-free survival in the high-risk group (53% vs. 90%) [161]. These observations highlight the potential prognostic significance of MIF in lung adenocarcinoma; however, the predictive value of such signatures for lung adenocarcinoma recurrence requires validation in experimental and clinical studies.

Consistent with the in vitro and in silico findings, analyses of patient-derived lung tissues have shown that MIF was markedly upregulated in NSCLC compared with normal lung epithelium [162], with particularly high expression observed in patients with squamous cell carcinoma, where it correlated with poorer prognosis [51]. Elevated levels of oxMIF were also detected in lung tumor tissues [57,153]. Moreover, MIF expression was higher in multiple primary lung adenocarcinomas than in single primary tumors, with both exceeding normal lung tissue levels, implicating MIF in the development of multifocal disease [156]. In contrast, a pilot study of 25 patients with newly diagnosed lung carcinoma versus 25 matched controls found no difference in serum MIF levels [163], highlighting the limited utility of circulating MIF as a biomarker for lung cancer when compared with tissue expression.

Elevated MIF expression has also been associated with aggressive features in lung cancers. For instance, in lung squamous cell carcinoma, a high proportion of MIF-positive tumor cells correlated with lymph node metastasis, shorter disease-free survival, and poorer cancer-specific outcomes [49]. Analysis of 370 NSCLC tumor samples revealed an inverse correlation between MIF and miR-451 expression, with elevated MIF linked to advanced disease and poorer prognosis [164]. In NSCLC tissues from 87 patients, MIF exhibited a bimodal pattern, with one group comparable to normal tissue and another markedly elevated [165]. In this cohort, high MIF levels were associated with increased angiogenic chemokines and VEGF, enhanced tumor growth and vessel density, poorer survival, and higher risk of recurrence after tumor resection [165]. This bimodal expression pattern could potentially be driven by underlying MIF polymorphisms in the patients, which influence MIF expression profiles; however, this possibility was not investigated in the study.

Increased MIF has also been demonstrated to confer resistance to chemotherapeutic agents. Cisplatin-resistant NSCLC tumors, as well as cisplatin-resistant A549 and H460 cells, exhibited elevated MIF expression, enhanced self-renewal, and M2 macrophage polarization, suggesting that MIF, together with markers such as proto-oncogene tyrosine-protein kinase Src and CD155, contributes to chemoresistance and tumor progression [166]. Similarly, in gefitinib-resistant lung adenocarcinoma patients, serum exosomal MIF levels were higher than the sensitive cases and positively correlated with tissue inhibitor of metalloproteinase-1 (TIMP1) expression [167]. Further in vitro studies showed that MIF mRNA was markedly upregulated in gefitinib-resistant A549 and PC9 cells, and M2 macrophage–derived exosomal TIMP1 was shown to promote gefitinib resistance [167]. Conversely, a retrospective study reported no association between serum MIF levels and chemotherapy response in NSCLC and SCLC patients [168], demonstrating that circulating MIF is less informative for patient identification and prediction of treatment response than tissue or exosome-derived MIF.

Beyond MIF, its receptor CD74 was strongly expressed in NSCLC specimens and frequently co-localized with MIF, a pattern associated with elevated angiogenic chemokines and increased tumor vascularity [169]. In lung adenocarcinoma tissues, CD74 expression was higher in tumors compared with adjacent normal tissue and correlated with lymph node metastasis and advanced TNM (tumor–node–metastasis) stage [170]. In vivo, TNF-α-driven inflammation in a urethane-induced lung adenocarcinoma mouse model upregulated MIF in macrophages and CD74 in tumor cells, while TNF-α blockade reduced their expression and tumor nodules, underscoring the TNF-α–MIF–CD74 axis in tumor progression [170].

Other in vivo studies further support the role of MIF and its receptors in promoting lung tumor growth and survival under experimental conditions. Jäger et al. demonstrated that A549 cells transduced to overexpress the MIF receptor CXCR4 exhibited increased proliferation and MIF production in vitro [171]. Intratracheal injection of these CXCR4-overexpressing cells into mice led to the highest lung tumor burden compared with mice receiving control or CXCR4-KO A549 cells. Bioluminescence and histological analyses revealed that treatment of mice with the MIF inhibitor ISO-1 significantly reduced tumor burden, highlighting the CXCR4–MIF axis as a driver of NSCLC progression in vivo [171]. While bioluminescence imaging provides a non-invasive method to monitor tumor growth, its accuracy can be limited by tissue depth and signal attenuation. High-resolution imaging techniques, such as micro-CT or MRI, could provide more precise tumor volume measurements, enabling better assessment of MIF-driven tumor progression and therapeutic responses.

Earlier in vivo work demonstrated that lung injury followed by intratracheal injection of LLC cells increased tumor growth in wild-type mice, with enhanced proliferation and reduced apoptosis, whereas MIF knockout mice exhibited neither injury-induced tumor growth nor alterations in proliferation or apoptosis, highlighting MIF’s essential role in linking tissue injury and repair to tumor progression [172]. Our previous work, as mentioned in Section 2.3, demonstrated that MIF’s tautomerase activity contributes to lung tumor progression in the LLC mouse model [77]. However, one limitation of this study was the use of a subcutaneous (ectopic) lung tumor model, where tumors develop outside their native lung environment, failing to replicate the lung’s unique stromal interactions and biomechanical forces, such as ventilation and perfusion dynamics [173,174]. These factors may influence treatment responses, highlighting the need for further studies to investigate MIF’s role, its tautomerase activity, and polymorphisms in orthotopic primary lung cancer models that more accurately reflect lung cancer biology.

In summary, evidence from in vitro, in silico, in vivo, and human tumor tissue studies supports a central role for MIF in lung cancer progression, angiogenesis, immune modulation, and therapeutic resistance. Elevated tumor tissue expression correlates with advanced disease stage and adverse clinicopathological features, supporting its potential prognostic relevance. In contrast, circulating serum MIF has demonstrated limited predictive value. This highlights a key translational limitation of circulating MIF as a reliable biomarker in lung cancer, whereas tumor tissue-based assessment may represent a more informative approach. Genetic determinants, such as high-expression CATT promoter polymorphisms, may warrant further investigation to determine whether they could serve as candidate stratification markers in lung cancer.

**Figure 3:**
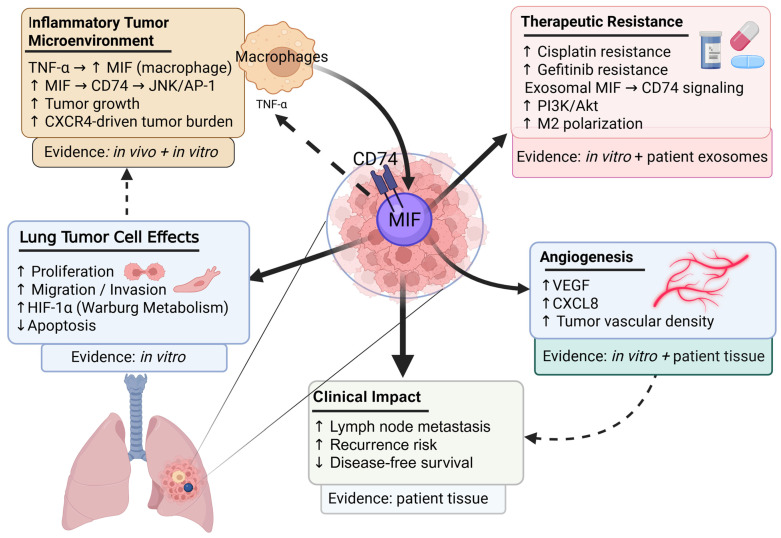
Macrophage migration inhibitory factor (MIF)-driven lung tumor progression and tumor microenvironment interactions. MIF acts as a mediator of lung tumor progression, in the inflammatory tumor microenvironment, tumor necrosis factor-alpha (TNF-α) increases macrophage-derived MIF, which signals through CD74 and promotes tumor growth. In lung tumor cells, MIF enhances proliferation, migration/invasion, hypoxia-inducible factor 1α (HIF-1α)-associated metabolic reprogramming and suppresses apoptosis. MIF also promotes angiogenesis via vascular endothelial growth factor (VEGF) and C-X-C motif chemokine ligand 8 (CXCL8) upregulation, contributes to cisplatin and gefitinib resistance through CD74–phosphatidylinositol 3-kinase/protein kinase B (PI3K/Akt) signaling and M2 polarization, and is associated with lymph node metastasis, recurrence risk, and reduced disease-free survival. Created in BioRender. Selo, M. A. (2026) https://BioRender.com/75jt8g3, accessed on 25 February 2026.

From a clinical perspective, MIF represents a potential therapeutic target, including antibody-based strategies, while the MIF–CD74 signaling axis may provide opportunities for rational combination approaches with immune checkpoint inhibitors. Furthermore, the association of MIF signaling with cisplatin and gefitinib resistance suggests that tumor MIF expression or activation of the MIF–CD74 pathway may represent candidate predictive biomarkers of response to chemotherapy or targeted therapy. Prospective validation will be essential to translate these observations into meaningful clinical utility. Figure 3 provides an integrated overview of tumor-intrinsic effects, microenvironmental interactions, therapeutic resistance, and the clinical impact of MIF in lung cancer.

## 7. Therapeutic Targeting of MIF

MIF is a key regulator of various cellular processes, and its dysregulation has been linked to a wide range of inflammatory and proliferative diseases. Thus, MIF is a promising therapeutic target, particularly in individuals with high baseline or inducible MIF expression, such as carriers of CATT6–8 alleles. MIF inhibitors can be generally divided into three main classes: small-molecule compounds, monoclonal antibodies, and peptide-based inhibitors [175].

### 7.1. Small-Molecule MIF Inhibitors

Small molecules targeting the MIF tautomerase active site or otherwise disrupting its function have demonstrated encouraging preclinical activity. Several such inhibitors have been identified using virtual screening techniques [70,176,177] and high-throughput activity-based assays [178,179]. Representative examples include ISO-1, the most widely used and commercially available research compound [180], 4-IPP [69,76], and SCD-19 [75,77]. These compounds primarily target the tautomerase site, which mediates MIF’s interaction with CD74, thereby attenuating downstream signaling. Beyond tautomerase-site inhibitors, several compounds have been reported to modulate MIF activity through alternative mechanisms. For instance, ebselen, a synthetic organoselenium compound that mimics the antioxidant enzyme glutathione peroxidase, disrupts the trimeric structure of MIF by promoting its dissociation into monomers, thereby impairing MIF–CD74 signaling [179]. Another compound, p425, a small-molecule allosteric inhibitor, binds to the surface of the MIF trimer, blocking its tautomerase activity, its interaction with CD74, and thereby its pro-inflammatory effects [181].

Ongoing studies have expanded the chemical diversity of small molecule MIF inhibitors to include oxadiazole, triazole, benzoxazole, benzopyran, and benzophenone derivatives, with improved potency and mechanistic diversity in preclinical models. For a detailed overview of these inhibitors, including their structure–activity relationships and mechanistic insights, readers are referred to a recent comprehensive review by Guo et al. [182]. Notable examples include the oxadiazole derivative IPG1576 which selectively inhibited MIF tautomerase activity and demonstrated oral bioavailability, safety, and in vivo efficacy in a murine pancreatic cancer model, reducing tumor growth and enhancing antitumor immunity [183]. In addition, a benzopyran (7-hydroxycoumarin) derivative compound inhibited MIF tautomerase activity at the nanomolar level and disrupted MIF–CD74 binding, resulting in reduced A549 cell proliferation in vitro [184]. However, this study did not include pharmacokinetic or toxicity assessments; therefore, comprehensive in vivo evaluation of metabolic stability, bioavailability, and safety profile assessment will be necessary to advance this scaffold toward the development of potent, clinically viable MIF inhibitors. Another remarkable example of genotype-selective inhibition is the phenazine derivative 1-methoxy-5-formyl-4,6,8-trihydroxyphenazine (CMFT), which blocked ICBP90 binding to the −794 CATT5–8 MIF promoter microsatellite. CMFT inhibited MIF transcription in a promoter length-dependent manner, selectively reducing MIF mRNA and protein in macrophages isolated from high-expression CATT7 mice, with minimal effect in CATT5 macrophages [185]. Notably, CMFT belongs to a class of compounds with poor in vivo metabolic profiles [186], which could limit its further clinical development. Nonetheless, its selective mechanism of MIF inhibition offers a valuable framework for refining this compound class and developing future precision-based MIF modulators for individuals with genetically high MIF expression.

Among many small-molecule MIF inhibitors developed, only a few have advanced to clinical evaluation. Ibudilast, which binds near, but not directly at, the N-terminal proline [187,188], has completed Phase I/II trials for alcohol use disorder, demonstrating acceptable safety but modest efficacy [189,190]. It was also evaluated in combination with temozolomide in a Phase 1b/2a study for glioblastoma (NCT03782415), where safety, tolerability, and preliminary efficacy were assessed, although detailed results have not yet been reported [191]. IPG1094, another small-molecule MIF antagonist, is undergoing a Phase 1/2 open-label, dose-escalation trial in patients with advanced solid tumors, including NSCLC, head and neck cancer, and glioma (NCT06212076), evaluating safety, tolerability, pharmacokinetics, pharmacodynamics, and initial anti-tumor activity, with results still pending [192]. These trials highlight the translational feasibility of advancing MIF-targeted small molecules into safe and effective human therapies, although broader clinical validation remains necessary.

Small-molecule inhibitors offer several translational advantages that complement biologic strategies in oncology. Due to their low molecular weight, many small-molecule agents are orally bioavailable, enabling convenient administration compared with the parenteral delivery required for monoclonal antibodies [193,194]. Their smaller size facilitates improved tissue and tumor penetration and allow access to intracellular targets that are less accessible to large biologic molecules [193,195]. In addition, small molecules benefit from well-established medicinal chemistry optimization pathways that permit systematic refinement of potency, selectivity, and pharmacokinetic properties during drug development [194,195]. Compared with complex biologics, small molecules generally involve simpler chemical synthesis and lower manufacturing complexity, which can translate into reduced production costs and potentially shorter development timelines [196]. While challenges such as resistance and off-target effects remain, these advantages provide a rationale for the continued prioritization of small-molecule approaches in cancer drug development.

In summary, although promising results have been observed in cellular and animal models, most small-molecule MIF inhibitors remain in early developmental stages. The broad range of MIF’s biological activities means that its inhibition may lead to adverse effects or toxicity, highlighting the need for thorough safety evaluation. Additional challenges include limited pharmacokinetic and toxicological data, as well as species differences in preclinical models, which hinder clinical translation. Continued efforts to optimize selectivity, metabolic stability, and safety, together with mode-selective approaches targeting specific MIF–receptor interactions, will be essential to fully realize the therapeutic potential of these compounds in inflammatory, immune, and cancer-related diseases.

### 7.2. Monoclonal Antibodies and Nanobodies

Monoclonal antibodies targeting MIF have been developed to neutralize its pro-inflammatory and pro-tumorigenic functions. These antibodies bind either native or oxidized form of MIF, thereby preventing interaction with its receptor CD74 and downstream signaling. In parallel, antibodies directed against CD74 itself have also been explored to disrupt MIF–CD74 signaling and inhibit MIF-mediated immune and tumor-promoting effects. Preclinical studies demonstrated therapeutic efficacy of anti-MIF antibodies in multiple disease models. In mice, it delayed arthritis onset, reduced its frequency [197], and lowered inflammatory cytokine levels and synovial inflammation [198]. In a rat model of anti-glomerular basement membrane disease, a neutralizing anti-MIF monoclonal antibody reduced proteinuria, preserved renal function, and attenuated histological signs of inflammation [199].

The human anti-oxMIF monoclonal antibody, imalumab, has undergone Phase I and early Phase IIa clinical trials in patients with advanced solid tumors, either alone or in combination with standard treatments [154,200]. The antibody was generally well tolerated, with allergic alveolitis as the only dose-limiting toxicity, and target engagement was confirmed, although objective tumor responses were limited, disease stabilization was observed in approximately one-quarter of treated patients [154,200]. These results underscore the need for further studies to optimize dosing, assess combination strategies, and clarify the therapeutic potential of MIF-targeted antibodies in oncology. Similarly, the humanized anti-CD74 antibody milatuzumab was evaluated in a Phase 1b trial in patients with moderate SLE, where it demonstrated reductions in disease activity and acceptable tolerability, with only mild injection-site or flu-like reactions observed in the initial cohort [201,202]. In oncology, a Phase I study in B-cell non-Hodgkin lymphoma and chronic lymphocytic leukemia showed acceptable safety but no objective tumor responses, likely due to rapid clearance caused by binding to CD74 on normal immune cells, which limited sustained drug levels in circulation [203]. However, broader randomized studies have not yet been conducted, and advancement into late-stage clinical development remains outstanding. Milatuzumab has also received FDA orphan drug designation for the treatment of multiple myeloma, but it has not yet been approved for this indication [204]. Notably, preclinical studies combining milatuzumab with the anti-CD20 antibody rituximab showed promising efficacy in mantle cell lymphoma model [205], highlighting potential for combination therapies in malignancies.

Nanobodies, single-domain antibody fragments, represent an emerging class of biologics with high solubility, stability, and superior tissue penetration compared to conventional antibodies [206]. Anti-MIF nanobodies with nanomolar affinity for murine and human MIF have been developed and shown therapeutic potential in models of septic shock and inflammatory organ damage [207]. Additional variants, including constructs with extended half-life, have provided more options for targeting MIF and evaluating improved stability and efficacy in preclinical studies [207]. To our knowledge, however, these anti-MIF nanobodies have so far been tested only in acute inflammatory disease models.

In conclusion, while antibodies and nanobodies offer high selectivity and potent engagement of extracellular MIF or its receptor interactions, challenges such as high production costs, and limited intracellular access hinder broader therapeutic development. Ongoing efforts to improve delivery and optimize these molecules may help address these limitations and make MIF-targeted biologics more feasible for clinical use.

### 7.3. Peptide Inhibitors

Peptide inhibitors represent a third class of therapeutics that may expand strategies for targeting MIF. For example, the novel chimeric peptide DRα1-MOG-35–55, which combines the HLA-DRα1 domain with the myelin oligodendrocyte glycoprotein 35–55 (MOG) peptide, binds to CD74 on monocytes and blocks MIF activity. This results in reduced tissue damage and improved symptoms of autoimmune encephalomyelitis in a mouse model [208]. The related inhibitor RTL1000, a fusion construct of DRα1 and DRβ1 domains with MOG-35–55 peptide (DRα1β1-MOG-35–55), also blocks MIF binding to CD74 and was well tolerated in a Phase 1 clinical trial for multiple sclerosis, highlighting its potential to promote targeted immunoregulation and central nervous system repair without inducing widespread immunosuppression [209,210]. The 17-amino acid peptide C36L1 also disrupts MIF–CD74 interactions on monocytes and dendritic cells, restoring antitumor immune responses by enhancing CD8^+^ T-cell activity in a metastatic melanoma model [40]. Finally, the synthetic peptides MIF-(40–49) and MIF-(47–56), which mimic the N-like loop of parent MIF, competitively inhibit MIF binding to CXCR2 resulting in reduced monocyte arrest on aortic endothelial cells in vitro and MIF-dependent monocyte adhesion to atherosclerotic carotid arteries in vivo [211], highlighting their potential to limit vascular inflammation and atherogenesis.

These therapeutic approaches illustrate the expanding spectrum of MIF-targeted interventions across small molecules, antibodies, and peptide-based strategies. Numerous compounds have been described, spanning distinct mechanistic classes and stages of development. Table 3 provides a representative overview of selected agents from each major mechanistic class, including compounds that have advanced to clinical trials as well as key preclinical inhibitors that have guided current therapeutic development. The translational challenges associated with these strategies are discussed in the following subsection.

### 7.4. Translational Challenges and Therapeutic Considerations

Despite encouraging preclinical results, several challenges arise when targeting pleiotropic cytokines such as MIF. MIF participates in innate and adaptive immune responses as well as broader stress-related signaling pathways, and sustained inhibition may therefore disrupt immune homeostasis and physiological inflammatory regulation [212]. Cytokine network redundancy represents an additional limitation. MIF interacts with multiple inflammatory mediators and signaling cascades, and compensatory mechanisms within the cytokine network may limit the efficacy of single-target interventions [60,213,214].

Pharmacokinetic and safety considerations also remain relevant. Some small-molecule inhibitors exhibit suboptimal physicochemical and pharmacokinetic properties, including limited metabolic stability and bioavailability [177,215]. Antibody-based approaches may be influenced by systemic clearance, aggregation-related properties, and antigen sink effects, particularly when targeting broadly expressed receptors such as CD74 [169,203,216]. Across therapeutic classes, comprehensive toxicological evaluation remains essential to ensure sustained target engagement and clinical durability.

More broadly, inflammation-targeting strategies in oncology have shown variable clinical benefit. While immune checkpoint blockade has transformed lung cancer treatment, the direct modulation of individual cytokines has not consistently produced durable antitumor responses across solid tumors [214]. In this context, MIF-targeted therapies may be better positioned within rational combination strategies rather than as monotherapy.

Accordingly, development may benefit from biomarker-guided identification of high MIF-expressing tumors or high-expression genetic variants, in which MIF-targeted strategies could potentially enhance existing therapies, an approach that would require prospective clinical validation. At the same time, given MIF’s pleiotropic roles in immune regulation and host defense, long-term systemic inhibition will require careful evaluation of safety and potential unintended immunomodulatory effects.

## 8. Conclusions and Future Perspectives

Accumulating evidence establishes MIF as a central mediator of inflammation and tumor progression across multiple malignancies. In lung cancer specifically, MIF has been associated with tumor progression, adverse prognostic features, immune modulation, and resistance to chemotherapy and targeted agents. Emerging translational strategies—including isoform-selective targeting of oxMIF, next-generation antibody engineering, nanobody-based approaches, and biomarker-guided patient stratification—reflect growing efforts to enhance therapeutic precision. At the same time, important hypotheses remain in lung cancer. High-expression MIF genetic variants have been associated with inflammatory and autoimmune diseases and several solid tumors; however, their role in lung cancer susceptibility and therapeutic response remains hypothetical. Clarifying these relationships will be essential to determine whether MIF inhibition can be integrated into personalized treatment approaches. These translational priorities and open questions are explored in detail below.

Despite these advances, no MIF-targeted therapy has yet achieved regulatory approval. Although several small molecules, antibodies, and peptide-based inhibitors have advanced to early-phase clinical evaluation, successful clinical translation remains limited. Greater emphasis is therefore needed on the development of clinically viable inhibitors with improved selectivity, pharmacokinetic properties, and safety profiles. Future research should focus on optimizing these characteristics and evaluating MIF-targeted strategies in cancer, particularly lung cancer, either as monotherapy or in rational combination with immunotherapy or standard chemotherapy.

Building on the need for more selective and clinically viable MIF inhibitors, selectively targeting oxMIF represents an emerging and therapeutically attractive strategy. Isoform-selective inhibition could attenuate pathological MIF signaling while preserving the homeostatic functions of reduced MIF in healthy tissues, thereby potentially reducing the risk of adverse effects. The feasibility of this approach is supported by early clinical evaluation of the anti-oxMIF antibody imalumab [154]. Second-generation bioengineered anti-oxMIF antibodies, such as ON203 and ON103, retain isoform specificity, exhibit improved biophysical properties, and show robust in vitro activity [217]. Notably, ON203 has demonstrated antitumor efficacy in a mouse preclinical model, significantly inhibiting the growth of human PC3 prostate cancer xenografts [217]. However, to our knowledge, no small-molecule MIF inhibitors are oxMIF-specific, making the development of such compounds an unmet challenge and opportunity to improve the precision of MIF-targeted therapies.

Expanding on these molecular interventions, nanobody-based therapeutics have shown considerable promise in cancer due to their small size, high stability, strong target affinity, and superior penetration of solid tumors [218,219]. Their remarkable stability under proteolytic conditions and acidic pH enables them to remain functional within the hostile tumor microenvironment, while their ability to recognize specific and cryptic epitopes allows precise targeting of tumor- and immune-associated factors [220,221]. Given the established role of MIF in cancer progression in general, and in lung cancer specifically, MIF may serve as a suitable epitope target for nanobody-based therapeutic strategies. However, anti-MIF nanobodies remain largely unexplored in oncology, including lung cancer. Further investigation of anti-MIF nanobodies could enable more precise modulation of the tumor microenvironment and open new avenues for targeted intervention in lung cancer, particularly in individuals with genetically high MIF expression.

Beyond molecular design, the use of nanocarriers to deliver targeted MIF therapies directly to affected organs represents an additional area for future studies. For example, inhalable or nebulized delivery systems could allow localized administration of non-encapsulated or nanocarrier-formulated anti-MIF therapies directly to the lungs in patients with lung cancer, potentially enhancing therapeutic efficacy while minimizing systemic exposure and off-target effects. Using nanomedicine approaches, these delivery systems could improve the distribution of MIF inhibitors, enable controlled release in response to tumor-specific signals such as acidic pH or enzymatic activity, and enhance their impact on cancer cells and the local tumor microenvironment, including immune and stromal components within lung tumors.

In addition, future studies should aim to validate MIF-related biomarkers to support patient stratification and therapeutic decision-making. Given the limited predictive value of circulating serum MIF, research should investigate tissue- or exosome-based MIF levels and MIF CATT genotyping as potential biomarkers to stratify patients with lung cancer and other MIF-driven diseases, and to guide therapeutic decisions, with further studies needed to confirm their clinical utility and ability to monitor treatment responses.

Another potential avenue for future investigation is the mechanistic interplay between MIF, high-expression CATT6–8 alleles, and key oncogenic pathways such as p53. Specifically, elucidating whether lung cancer patients carrying these high-expression CATT alleles and wild-type p53 exhibit poorer prognosis and whether elevated MIF levels attenuate the tumor-suppressive activity of p53 could enhance our understanding of disease mechanisms and guide more effective therapies. Furthermore, assessing whether high-expression CATT alleles predict resistance to PD-1/PD-L1 checkpoint inhibitors could provide insights into the genetic basis of therapeutic response.

In a related context, the MIF CATT polymorphism has been extensively studied in inflammatory and autoimmune diseases, where individuals carrying high-expression alleles may benefit from MIF inhibition, including steroid-sparing strategies for treatment-resistant or glucocorticoid-dependent patients [175]. Extending these insights into cancer, particularly lung cancer, is a critical next step. Although MIF has been investigated in various cancers, the specific impact of high-expression MIF alleles on lung cancer biology remains unexplored, representing a critical knowledge gap. These high-expression alleles are relatively common in the population, and defining their influence on lung cancer initiation, progression, and metastasis could help identify patients genetically predisposed to elevated MIF levels who may preferentially benefit from MIF-targeted therapies. Future studies should evaluate MIF inhibition specifically in carriers of these alleles, with the goal of advancing personalized medicine approaches that tailor treatment to patients genetically primed for high MIF expression.

## Figures and Tables

**Table 1:** Reported cell types expressing macrophage migration inhibitory factor (MIF), including experimental context (in vitro/in vivo; species or model) and key conditions associated with its induction.

Cell Type	Context (In Vitro/In Vivo; Species/Model)	Inducers/Conditions Associated with Macrophage Migration Inhibitory Factor (MIF) Expression	Reference(s)
Macrophages (including alveolar macrophages)	In vitro human and murine macrophage cultures; ex vivo human alveolar macrophages and in vivo lung inflammation models	Constitutive expression and release; upregulated by lipopolysaccharide (LPS), tumor necrosis factor-alpha (TNF-α), interferon-γ, and during pulmonary inflammatory conditions	[31,34,35,36]
T lymphocytes	Activated mouse and human T-cells (in vitro and in vivo models)	Express and secrete MIF upon activation; supports proinflammatory T-cell responses and T-cell-dependent antibody production	[34,37,38]
B lymphocytes	Human peripheral blood B-cells (ex vivo, flow cytometry; sepsis); in vitro/ex vivo memory B-cell studies	Express intracellular MIF and secrete MIF under immune activation	[34,35,39]
Dendritic cells	In vitro and in vivo immune models; tumor microenvironment studies	Reported to express MIF; functionally regulated via MIF–CD74 signaling in immune and tumor contexts	[34,40,41]
Epithelial cells (including lung epithelial cells)	Constitutive expression in epithelial barrier tissues (including lung); in vitro human epithelial carcinoma cell lines; in vivo human tumor tissues	Baseline epithelial expression; increased expression in lung cancer and under hypoxic conditions	[34,42,43,44]
Endothelial cells	In vitro human vascular endothelial cells; in vivo murine inflammatory models	Constitutive expression; upregulated by inflammatory stimuli (e.g., LPS, TNF-α); implicated in leukocyte recruitment/extravasation	[34,44,45,46]
Tumor cells (e.g., non-small cell lung cancer (NSCLC))	In vitro human lung cancer cell lines; in vivo murine tumor models; human tumor tissues	Oncogenic stress, hypoxia, and tumor microenvironment-associated signaling	[44,47,48,49,50,51]

**Table 2:** Associations of macrophage migration inhibitory factor (MIF) promoter polymorphisms (−173 G/C and −794 CATT5–8) with disease susceptibility and severity in inflammatory, autoimmune, and malignant disorders.

Disease or Condition	Polymorphism(s)	Associated Allele	Reported Association	Reference(s)
Rheumatoid arthritis	−173 G/C and −794 CATT5–8	−173 C and CATT7 alleles	Increased susceptibility and Increased severity	[80,92,105]
		CATT5 allele	Low disease severity	[81]
Inflammatory polyarthritis	−173 G/C and −794 CATT5–8	−173 C CATT7 alleles	Increased susceptibility	[106]
Systemic lupus erythematosus	−173 G/C and −794 CATT5–8	−173 C and CATT7 alleles	−173 C and CATT7 are associated with increased end-organ damage, but their relationship with disease incidence is controversial, as studies report both increased and decreased risk.	[83,93,107]
Juvenile idiopathic arthritis	−173 G/C	−173 C allele	Increased susceptibility	[79]
Ulcerative colitis	−173 G/C	−173 C allele	Increased risk	[108]
Autoimmune hepatitis	−173 G/C	−173 CC/GC genotypes	Higher alanine aminotransferase levels and greater steroid requirements were observed compared with the GG genotype.	[109]
Psoriasis	−173 G/C	−173 C allele	Associated with male and late-onset psoriasis	[110]
Pulmonary tuberculosis	−173 G/C	−173 C allele	Increased susceptibility, particularly in Asians.	[90]
Coronary heart disease	−173 G/C	−173 C allele	Increased risk in Arab and Asian populations	[88,89]
Pneumococcal meningitis	−173 G/C and −794 CATT5–8	−173 C and CATT7 alleles	Increased morbidity and mortality	[111]
Cystic fibrosis	−794 CATT5–8	CATT5 allele	Milder disease, reduced *Pseudomonas* colonization, and slower forced expiratory volume in 1 s (FEV1) decline in CATT5 than CATT6–8 carriers.	[74,94]
COVID-19 patients and mouse model	−794 CATT5–8	CATT7 allele	Increased disease severity but reduced susceptibility in comparison with CATT5	[100]
Asthma (preclinical mouse model)	−794 CATT5–8	CATT7 allele	More pronounced airway inflammation in CATT7 than in CATT5	[101,103]
Cancer (various types)	−173 G/C	−173 C (G/C + C/C genotypes)	Increased overall cancer risk	[91]
Breast cancer	−173 G/C and −794 CATT5–8	−173 C and CATT7 alleles	Higher circulating macrophage migration inhibitory factor (MIF) levels without increased susceptibility	[112]
Cutaneous squamous cell carcinoma	−173 G/C and −794 CATT5–8	5 C (CATT5/−173 C) and 7 G (CATT7/−173 G) haplotypes	Linked to increased susceptibility and higher circulating MIF levels	[99]
Rectal cancer	−173 G/C and −794 CATT5–8	−173 C and longer (CATT6–8) repeats	Elevated serum MIF levels and increased susceptibility	[95]
Non-cardia gastric cancer	−173 G/C and −794 CATT5–8	−173 C and longer (CATT6–8) repeats	Increased susceptibility to gastric cancer, synergistic effect with *H. pylori* infection.	[96]
Hepatocellular carcinoma	−173 G/C	−173 C (CC and GC) genotypes	Associated with increased susceptibility, poor prognosis and metastasis	[113]
Early-stage cervical cancer	−794 CATT5–8	CATT7 allele	Associated with increased susceptibility.	[97]
Prostate cancer	−173 G/C and −794 CATT5–8	G/C and C/C genotypes and CATT7 allele	G/C and C/C genotypes associated with increased incidence; CATT7 allele associated with increased incidence and higher risk of recurrence	[98]
Melanoma	−173 G/C	−173 C (C/C) genotype	More frequent in patients than controls, suggesting increased susceptibility	[104]

**Table 3:** Representative therapeutic strategies targeting macrophage migration inhibitory factor (MIF). Selected small-molecule inhibitors, antibodies, and peptide-based approaches are summarized, highlighting mechanism of action, key findings, development status, and reported limitations.

Agent	Class/ Mechanism	Key Findings	Clinical Status	Key Limitations/Adverse Effects	Reference(s)
ISO-1	Small molecule tautomerase inhibitor	Reduced inflammatory cytokine production and decreased lung tumor growth in murine models	Preclinical	No clinical safety data; limited potency and pharmacokinetic (PK) characterization	[73,171]
SCD-19	Potent small molecule tautomerase inhibitor	Reduced pulmonary inflammation and markedly suppressed lung tumor growth in murine models	Preclinical	No clinical safety or PK data	[75,77]
Ibudilast	Small-molecule allosteric inhibitor of macrophage migration inhibitory factor (MIF)	Demonstrated neuroimmune modulation in alcohol use disorder; evaluated in combination with temozolomide for glioblastoma	Phase I/II (alcohol use disorder); Phase 1b/2a (glioblastoma)	Gastrointestinal symptoms, headache, fatigue, dizziness; modest efficacy in alcohol use disorder; limited oncology efficacy data	[189,190,191]
IPG1094	Small-molecule MIF antagonist	Under evaluation for safety and preliminary antitumor activity in advanced solid tumors including non-small cell lung cancer (NSCLC)	Phase I/II (ongoing)	Clinical outcomes and safety profile pending publication	[192]
1-methoxy-5-formyl-4,6,8-trihydroxyphenazine (CMFT)	Genotype-selective small molecule targeting the CATT microsatellite–ICBP90 axis	Selectively reduced MIF expression in macrophages isolated from high-expression CATT7 mice	Preclinical	Poor metabolic stability; no clinical safety data	[185,186]
Imalumab	Anti-oxMIF monoclonal antibody	Stable disease in a subset of advanced solid tumor and metastatic colorectal cancer patients	Phase I–IIa	Fatigue, nausea/vomiting, infusion reactions; dose-limiting allergic alveolitis; modest antitumor activity	[154,200]
Milatuzumab	Anti-CD74 monoclonal antibody	No objective tumor responses in Phase I B-cell malignancies; preliminary reduction in disease activity in Phase Ib systemic lupus erythematosus (SLE)	Phase I (oncology); Phase Ib (SLE)	Infusion reactions, anemia, lymphopenia, neutropenia, thrombocytopenia; rapid clearance due to peripheral antigen sink; limited data in solid tumors	[201,202,203]
DRα1β1-MOG-35–55 (RTL1000)	Peptide construct blocking MIF–CD74 interaction	Reduced inflammation in autoimmune models; well tolerated in Phase I trial in multiple sclerosis	Phase I	Not evaluated in oncology	[209,210]
C36L1	Peptide inhibitor disrupting MIF–CD74 signaling	Restored antitumor immune responses in metastatic melanoma models	Preclinical	No clinical translation: toxicity profile not established	[40]
MIF-derived peptides (40–49; 47–56)	Peptides inhibiting MIF–CXCR2 interaction	Reduced monocyte recruitment and vascular inflammation in vitro and in vivo	Preclinical	Limited translational development; no clinical safety data	[211]

## Data Availability

No new data were created or analyzed in this review. Data sharing is therefore not applicable.

## Abbreviations

The following abbreviations are used in this manuscript:

4-IPP	4-iodo-6-phenylpyrimidine
AP1	Activator Protein 1
AP4	Activator Protein 4
CMFT	1-methoxy-5-formyl-4,6,8-trihydroxyphenazine
cPLA2	Cytosolic Phospholipase A2
CXCL8	C-X-C motif Chemokine Ligand 8
CXCR	C-X-C Chemokine Receptor
EMT	epithelial-to-Mesenchymal Transition
ERK	Extracellular Signal-Regulated Kinase
FEV1	Forced Expiratory Volume in 1 s
HIF-1α	Hypoxia-Inducible Factor 1α
JNK	c-Jun N-terminal Kinase
LLC	Lewis Lung Carcinoma
LPS	lipopolysaccharide
MAPK	Mitogen-Activated Protein Kinase
MIF	Macrophage Migration Inhibitory Factor
MOG	Myelin Oligodendrocyte Glycoprotein
NF-κB	Nuclear Factor kappa B
NSCLC	Non-Small Cell Lung Cancer
oxMIF	oxidized MIF
PD-1	Programmed Cell Death Protein-1
PD-L1	Programmed Death-Ligand-1
PI3K/Akt	Phosphatidylinositol-3-Kinase/Protein Kinase B
RA	Rheumatoid Arthritis
rhMIF	recombinant human MIF
SCLC	Small cell Lung Cancer
SLE	Systemic Lupus Erythematosus
SNP	Single-Nucleotide Polymorphism
TIMP1	Tissue Inhibitor of Metalloproteinase-1
TNF-α	Tumor Necrosis Factor-alpha
VEGF	Vascular Endothelial Growth Factor
